# Importance of the relationship between sinus cycle length and junctional rhythm cycle length (occured during radiofrequency ablation) in predicting the successful modification of the slow pathway in Atrioventricular Nodal Re-entrant Tachycardias

**Published:** 2008-08-01

**Authors:** Javier Jimenez-Candil, Jose Luis Morinigo, Claudio Ledesma, Victor Leon, Candido Martín-Luengo

**Affiliations:** Department of Cardiology, University Hospital, Salamanca, Spain

**Keywords:** Atrioventricular nodal re-entry  tachycardia, Ablation, Junctional ectopy

## Abstract

**Background:**

In atrioventricular nodal re-entrant tachycardias (AVNRT), the achievement of Junctional Rhythms (JR) during Radiofrequency Ablation (RF) is a sensitive but non-specific marker of success. Our aim is to analyze prospectively the predictors of non-inducibility of AVNRT, focusing on the characteristics of the JR.

**Methods:**

We included 75 patients with reproducibly inducible AVNRT. Ablation was performed following an electro-anatomical approach. After each application, the induction protocol was repeated.

**Results:**

A total of 341 applications were performed. Although the achievement of ≥1 JR was necessary to obtain the non-inducibility, and the cumulative number of junctional beats (CJB) was higher in effective applications, no CJB cut-off was associated with a success rate higher than 75%. After the observation of a significant correlation between the sinus cycle length (CL) pre-RF and the CL of the JR (JR-CL) (c=0.52; p<0.001), the sinus CL pre-RF/JR-CL ratio (CL-ratio) adequately differentiated the successful vs. unsuccessful applications: 1.41±0.23 vs. 1.17±0.2 (p<0.001). In a multivariate analysis, a CBJ  11 (p<0.001) and a CL-ratio 1.25 (p<0.001) were found to be the only independent predictors of success. The combination of ≥ 11 of CJB with a CL ratio ≥ 1.25 achieved non-inducibility in 97% of our patients.

**Conclusion:**

1) The specificity of the occurrence of JR as a marker of the successful ablation of AVNRT is increased by the CL-ratio. 2) The achievement of ≥ 11 of CJB with a CL ratio ≥ 1.25 predicts non-inducibility in almost all patients.

## Introduction

Atrioventricular nodal re-entrant tachycardia (AVNRT) is the most common cause of paroxysmal supraventricular tachycardias [1]. Selective radiofrequency catheter ablation (RF) of the slow AV nodal pathway has become the first-line curative treatment mode in patients with AVNRT [2-5] and relies on the non inducibility of the tachycardia as a criterion of success [6]. However, in 5 to 15 % of cases, AVNRT is not inducible or not reproducibly inducible during electrophysiological studies [7,8]. Although ablation of the slow AV nodal pathway is an accepted treatment for such patients [4,8], the optimal end-point of the treatment has not been well established because evidence of a junctional rhythm (JR) during RF is a non-specific marker of success [7,9].

Additionally, previous studies have not been able to pinpoint the clinical usefulness of the cycle length (CL) of JR in predicting the achievement of successful slow pathway ablation because the values observed during effective and ineffective energy applications overlap considerably [9]. We speculated that the CL of the JR (JR-CL) obtained during Radiofrequency Ablation would be related to the basal sinus CL; if this were the case, the ratio between sinus CL before application of RF and the mean CL of the JR (CL-ratio) could be a marker of successful applications. The purpose of the present study was hence to analyse prospectively the predictors of non-inducibility of AVNRT after applications of RF, focusing on the characteristics of the JR, the JR-CL, and the relationship of this latter with the sinus CL prior to RF ablation.

## Methods

### Characteristics of the study population

From July 2004 to March 2006, a total of 82 consecutive patients with clinically documented supraventricular tachycardia underwent an electrophysiological study and RF catheter ablation due to typical AVNRT at our institution. The study was approved by the Institutional Review Board of our institution. In 7 patients AVNRT was not reproducibly inducible; thus, our study population finally comprised 75 patients (mean age: 48?9; females: 84%; hypertension: 24%). No patient had major structural heart disease.

### Electrophysiological Study

After obtaining written informed consent, baseline electrophysiological study was performed with patients in the fasting unsedated state. All anthyarrhythmic drugs were discontinued at least five elimination half-lives prior to study. Three multipolar electrode catheters were introduced percutaneously via femoral veins and positioned under fluoroscopic guidance in the right atrium, the His bundle region, and the right ventricular apex. In some patients, a decapolar electrode catheter was introduced into the left antecubital vein and placed in the coronary sinus. Intracardiac electrograms were filtered at 30 to 500 Hz and simultaneously displayed with surface ECG leads I, II, V1 and V6 on a multichannel oscilloscope (Cardiolab 6.0, GE Medical Systems, Milwaukee, USA). A programmable stimulator (Model 5328, Medtronic, Inc) was used to deliver an electric stimulus with 2-ms duration at twice the diastolic threshold. Incremental pacing and programmed stimulation were performed in the right atrium and right ventricle to define anterograde and retrograde AV nodal conduction and to confirm that AVNRT was inducible. In 21 patients the tachycardia was not inducible in the baseline state; in these cases, isoproterenol (at graded doses from 1 to 4 μmg/min IV) was infused to facilitate induction. AVRNT was diagnosed using previously described criteria; intra-atrial re-entrant tachycardia and tachycardia incorporating a midseptal or paraseptal accessory pathway were excluded [10-13].

Junctional beats were identified based on the QRS configuration and duration identical to that of sinus beats and the absence of AV conduction from a preceding P wave. All episodes of JR during the applications of RF were analysed as regards the ablation site, the total number of junctional ectopic beats, the duration of JR, and the CL of the JR.

### Mapping and Ablation

Mapping and ablation were performed using a multipolar catheter (Marinr MC, Medtronic Inc., USA) with a distal electrode size of 4mm, following an anatomically guided approach [3]. Briefly, the triangle of Koch, extending from the coronary sinus ostium up to the His bundle region, is divided into three regions designated posterior, mid and anterior. The ablation catheter was placed along the tricuspid septal annulus down to the posterior aspect of the interatrial septum adjacent to the coronary sinus ostium (posterior zone), obtaining an AV electrogram ratio of 0.1 to 0.3. At the end of each RF delivery, the inducibility of AVNRT was tested and, if still inducible, a second RF pulse is delivered to an adjacent site with a higher AV ratio. In the case of further unsuccessful pulses, the catheter is moved towards a more mid and anterior position if necessary.

The RF energy was delivered while temperature was monitored at the catheter tip, which was limited to a maximum of 55 ºC, with a preset maximal power of < 45 W. The duration of the RF pulses was 30 seconds; in the presence of an accelerated JR (CL<450 ms), a JR without ventriculo-atrial conduction, a PR prolongation, a rise in impedance or a displacement of the catheter, the application was discontinued immediately. After each application, regardless of whether or not JR was observed, the induction protocol was repeated, including the infusion of isoproterenol in the cases in which it had been necessary previously.

### Location of the ablation site

Each ablation site was determined using three different fluoroscopic projections: right anterior oblique 20º, front view, and left anterior oblique 60º. Three areas of equal height between two principal reference points (inferior edge of the coronary sinus ostium and the detection site of distal His-bundle deflection) were determined: posterior, mid-septal and anterior.

### Follow-up

After the procedure, the patients were monitored for 24 hours prior to discharge. No antiarrhythmic drugs were prescribed. Follow-up information was obtained at the time of the clinical revisits, which took place 6 and 12 months after discharge. Whenever the patient had symptoms suggestive of tachycardia, they were advised to seek consultation from our arrhythmia clinic or contact their physicians to verify possible AVNRT recurrence.

### Definitions

- Reproducibly inducible tachycardia: Tachycardia was induced three or more consecutive times with the same protocol. 

- Non-inducible tachycardia: Post-ablation, AVNRT was no longer inducible with no more than a single atrial echo.

- Successful application: An application that achieved non-inducibility.

- Sinus Cycle Length: Mean cycle length of the 10 consecutive beats registered immediately before or after each RF application.

- Junctional Rhythm: The occurrence of ≥2 consecutive junctional beats.

- Junctional Rhythm Cycle Length: Average of the clycle length of all junctional rhythms or beats occurred during an application. In the case of isolated junctional beats, it was determined the coupling interval with the precedent sinus beat.

- Cumulative Junctional Beats: Number of junctional beats achieved from the first application to the present application.

- CL-ratio: Ratio of Sinus Cycle Length before RF/Junctional Rhythm Cycle Length.

### Statistical analysis

The statistical analysis was performed using the SPSS 11.5 for Windows (SPSS Inc., Chicago, Illinois). Normal and continuous variables were described using the mean and standard deviation, whereas categorical variables were summarized by the number of patients and percentage. In order to establish cut-off points of continuous variables with the best sensitivity and specificity, we determined the Receiver-Operating Characteristic Curves. Comparison of categorical variables was performed with the Chi-square test (or Fisher's exact test if n<5). Comparison of two normal (determined by the Kolgomorov-Smirnov test) and continuous variables was accomplished with Student's t test. Comparison of >2 continuous variables was performed using the ANOVA-test. Multivariate analysis was performed using the stepwise logistic regression test, including the variables with statistical significance in the univariate analysis. A P value < 0.05 was considered to represent a significant difference.

## Results

A total of 341 RF applications were performed (mean applications per patient: 4.6±2.4; median: 4; range: 1-13). In 190 applications (56 %) we achieved JR. The median of beats/application was 8. Non-inducibility was achieved in all cases, and no major complication occurred, in particular neither transient nor persistent second or third degree AV block. In all successful applications at least one JR was achieved. Table 1. No recurrences of AVNRT were recorded during a follow-up period of 1 year.

### Determinants of the JR-CL

**Figure 1:**
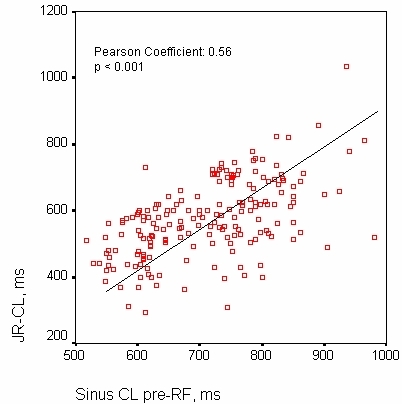
Diagram showing the correlation between the sinus CL and the JRCL

The mean JR-CL was 574±114 ms (median: 570 ms; interquartile range: 150 ms). The mean of the sinus CL before RF was 704±104 ms (median: 700). There was a positive and significant correlation between the sinus CL before RF and the mean CL of the subsequent JR achieved during the application (Pearson coefficient: 0.56; p<0.001). Figure 1. Applications preceded by a sinus CL of ≥700 ms were associated with a significantly higher mean JR-CL: 624±111 versus 512±84 (95 % CI of the difference: 83; 141; p<0.001).

The JR-CL was shorter in applications with a duration of the atrial electrogram ≥50 ms and in those performed after isoproterenol infusion. Other variables analysed were not related to any significant difference in the JR-CL. Table 2. In a multivariate analysis for accelerated JR (JRCL ≤550 ms), a sinus CL of <700 ms (OR: 4.3; 95% CI: 2.3-8.2; p<0.001) was the only independent predictor. The use of isoproterenol (OR: 1.47; 95% CI: 0.67-3.2; p=0.3) or a duration of the atrial electrogram of  ≥50 ms (OR: 1.9; 95% CI: 0.98-3.7; p=0.07) ceased to be statistically significant.

### Predictors of non-inducibility

Successful applications showed a more prolonged duration of  the atrial electrogram, a higher A/V voltage ratio, more junctional beats, more CJB, a longer duration of the JR, a shorter JR-CL, a higher CL-ratio,  and a higher frequency of the absence of VA conduction during the JR, and of mid-septal ablation sites. Table 1.

Two continuous variables showed an adequate correlation with non-inducibility (defined as an  area under the  ROC curve ≥ 0.8): CJB and the CL ratio. Table 3. The best cut-off point of the continuous variables with statistical significance were a CJB of ≥11, a CL ratio of ≥1.25, a duration of JR of ≥3.5 s, a JR-CL of ≤550 ms, a duration of atrial electrogram of ≤50 ms, and an A/V voltage ratio of ≥0.2. Their predictive values are shown in Table 3.

All of these variables, together with a mid-septal site of ablation and the absence of VA conduction during the JR, were associated with a significantly higher frequency of success in the univariate analysis. However, in the multivariate analysis, only two persisted as independent markers of non-inducibility: a CJB of  ≥11 and a CL ratio of  ≥1.25.  Table 4.

**Figure 2:**
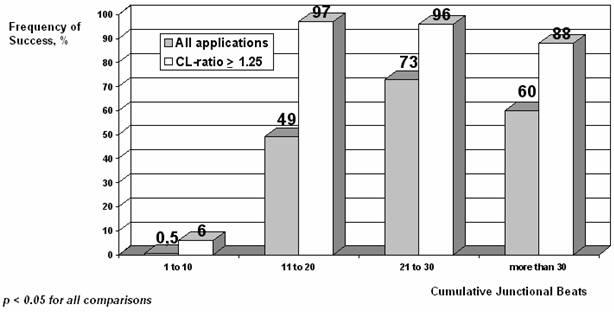
Bar diagram showing the frequency of success of the applications in relation to the number of CJB and the CL-ratio

Although the achievement of at least of one JR was necessary to demonstrate non-inducibility, no CJB was associated with a success frequency greater than 75 %. Nevertheless, a CL ratio of ≥1.25 significantly increased the positive predictive value for non-inducibility across the whole range of CJB values. Figure 2.

A total of 74 applications (performed in 70 patients) displayed a CJB of  ≥11 and a CL ratio of ≥1.25; of these, 70 (95 %) were successful. The success frequency was significantly higher in attempts with a CJB of ≥11 and a CL ratio of ≥1.25: 95% vs. 2% (OR: 13; 95% CI: 6-43; p<0.001). Applications with a CJB of ≥11 and a CL ratio of ≥1.25 had a sensitivity, specificity, negative predictive value and positive predictive value for non-inducibility of 93 %, 98 %, 98 % and 95 %, respectively. The four unsuccessful applications with a CJB of ≥11 and a CL ratio of ≥1.25 occurred in 2 patients (two in each). Thus, in 68/70 patients (97 %) the achievement of a CJB of ≥11 and a CL ratio of ≥1.25 was predictor of success. Focusing on the remaining five patients, four became non-inducible with a CJB of ≥11 and a CL-ratio of <1.25, and in one the number of CJB was 6 and the CL-ratio was 1.54.

In 33 patients (44 %) the successful application abolished the dual AV nodal pathway physiology. CL-ratio correlated with the probability of the dual AV nodal pathway physiology elimination (C-coefficient: 0.75; p<0.001). Clasifying the last applications into three groups according to the tertiles of CL-ratio (≤1.28; 1.29-1.42; ≤1.43), we found that the applications with higher CL-ratio were associated with a more elevated frequency of the abolition of the slow pathwaty function: 20% vs. 44% vs. 68% (p<0.001 for the trend). Other variables analysed did not show significant differences. Table 5.

## Discussion

The major finding of our study was that despite a positive and significant correlation between the sinus CL before RF and the mean of the JR-CL, the CL ratio increased the specificity and the positive predictive value of the amount of JR in predicting the acute success of slow pathway RF ablation.

### Junctional rhythm during slow pathway RF ablation: mechanism and implications

The precise mechanism responsible for the JR is not well established. Different hypotheses have been proposed, such as: a) the postganglionic release of noradrenaline from the sympathetic nerve endings, produced by the RF current, which may increase junctional automaticity [14]; b) an enhanced automaticity of heat-sensitive cells located close to the AV node in response to thermal effects of RF [15]; and, c) the thermal current conducted through specialized atrionodal fibbers to the AV node [16].

Although in some rare cases successful slow-pathway ablation is possible in the absence of junctional ectopy [17], the achievement of a JR  is a sensitive marker of success [9,18]. In the present study, the occurrence of a JR was universal in the effective applications; this also has been reported by others [9]. However, the specificity of this finding is limited; it appears in 25-65 % of unsuccessful attempts (9, 19). On the other hand, JR with a  CL of < 350 ms and absence of ventriculoatrial conduction during JR have been identified as markers of AV block [20,21].

### Predictors of Junctional Rhythm Cycle Length

In previous reports, the relationship between the JR-CL and several variables related to RF applications has been analysed: neither mid-septal ablation sites [19] nor the duration of the local atrial electrogram [22]  modified the JR-CL. We found that the JR-CL was related to the sinus cycle length, with a positive and significant correlation between both. Although in our study a duration of the atrial electrogram of ≥50 ms and the use of isoproterenol were also associated with shorter JR-CL, in the multivariate analysis the only independent predictor of JR-CL ≤550 ms was the sinus CL pre-RF. It is clear that the emergence of the JR always occurs with a CL shorter than the sinus rhythm, because JR with a CL longer than that of sinus rhythm would be suppressed by sinus node activity. Moreover, several works have shown that high levels of catecholamines enhance the automaticity of the nodal tissue [23] and facilitate the emergence of the JR during RF [24]; it is possible that the sinus CL, as an indicator of the level of activation of the sympathetic nervous system [25], might be associated with the JR-CL during RF applications through this mechanism.

### Specificity of the Junctional Rhythm in predicting successful ablation

Because the sensitivity of JR for successful slow pathway ablation is very high, it is useful to use the occurrence of  the JR as marker of effective RF applications. In general, the bursts of JR are significantly longer at successful sites [9,19,26], but the number of junctional beats objectified during effective and ineffective applications is considerably overlapped. Thus, the quantity of junctional ectopy during attempted RF treatment is not useful in predicting whether slow pathway ablation has been achieved [9]. Other authors have proposed that the total amount of JR is related to the total abolition of slow pathway conduction and may serve as a marker of success; unfortunately the specificity of this variable is very low [26]. In our study, the cumulative number of junctional beats (CJB) correlated with the non-inducibility; even though the correlation was adequate, no CJB presented a sufficiently high positive predictive value to be used as a predictor of success.

Other variables have been found to be independent predictors of successful RF attempts; thus the following may increase the specificity of the JR as marker of success: the ablation site (mid-septal rather than posterior) [19], the duration of  the atrial electrogram (including slow pathway potentials when present) [19] and a higher ablation temperature [27].

**Figure 3:**
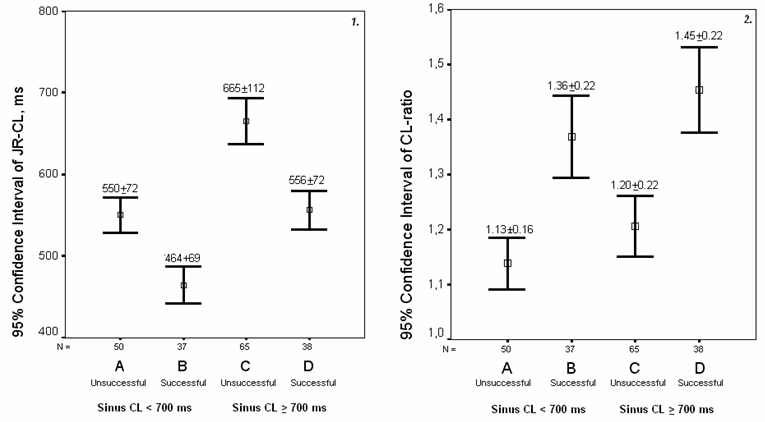
Scatter plot showing the JR-CL and the CL ratio of the applications when they were classified by the Sinus Cycle Length pre-RF and the result of the application. ***Panel 1:*** A vs. D: 95 % Confidence Interval of the Difference: -34; 42; p=0.8. B vs. C: 95 % Confidence Interval of the Difference: 85; 135; p<0.001. ***Panel 2:*** A vs. D: 95 % Confidence Interval of the Difference: 1.08; 1.34; p<0.001. B vs. C: 95 % Confidence Interval of the Difference: 0.12; 0.48; p=0.008.

During the application of RF, the JR is thought to be a marker of thermal injury, a shorter JR-CL has been associated with a high degree of lesion of the AV node [21,28]. This may have two practical consequences. Firstly, it has been demonstrated that a JR with a CL under 350 ms is a predictor of conduction block [21]. Secondly, the JR-CL could increase the specificity of the occurrence of JR for predicting the successful modification of the slow pathway with RF, as exemplified by either tachycardia non-inducibility or abolition of the dual AV nodal pathway physiology. This point has not yet been clarified. In several studies the effective applications did not differ in JR-CL compared with the junctional ectopy that occurred during failed attempts [9,19]; in other, the JR-CL was significantly shorter in successful applications (the authors only reported a univariate analysis), although with limited clinical value [29]. In the present study the JR-CL of the successful attempts was also shorter but, after adjusting for other significant variables, particularly the CL-ratio, the JR-CL was not an independent marker of success. The explanation of this finding could lie in the significant correlation found between the sinus CL and the JR-CL, which produces produced an important overlap between the JR-CL of effective applications preceded by a larger sinus CL and ineffective applications preceded by a shorter sinus CL. Figure 3.

In our series, the CL-ratio had the best correlation with the probability of success, avoiding the overlap of JR-CL between effective and ineffective applications. Its best cut-off point (a CL ratio of ≥1.25) increases significantly the specificity and positive predictive value for non-inducibility of the different ranges of CBJ. The combination of a CJB of ≥11 and a CL-ratio of ≥1.25 maintained a higher sensitivity, had a positive predictive value for success of 95 %, and achieved non-inducibility in 97% of our patients.

Finally, up to 40 % of patients have residual slow pathway function after successful ablation of AVNRT [2,30]; our data show that, among successful applications, the higher the CL-ratio the more probable the elimination of the dual AV nodal pathway physiology.

### Clinical implications

The major clinical implication of the present study is that, in an electro-anatomical approach, the combination of a CJB of ≥11 and a CL ratio of ≥1.25 is useful to assess when the successful slow pathway modification has been achieved. This could allow to test the inducibility more efficiently, avoinding unnecessary applications and shortening the duration of the procedures. In addition, since in 5-15 % of patients with documented paroxysmal supraventricular tachycardia the tachycardia is non-inducible (or non-reproducibly inducible) [7,8], and since for these patients the slow pathway ablation is accepted as treatment [4,8], the achievement of a CJB of ≥11 and a CL-ratio of ≥1.25 could be used as the end-point of the RF.

## Study Limitations

-The lower number of patients could decrease the statistical power of our findings.

-Since the location of the ablation sites was determined exclusively with fluoroscopic and electrical references, its accuracy might not be optimal.

-The number of cumulative junctional beats (CBJ) included all junctional beats occurred from the first to the present application. This could decrease the value of the parameters assessed in the present application, since there is an influence from previous applications. This is a difficulty common to all studies including several RF applications in a single patient.

-The analysis was performed taking in consideration isolated junctional beats and junctional rhythms together. This did not allow to determine differences between both.

## Conclusions

The specificity of the occurrence and amount of the JR as marker of successful slow pathway ablation is insufficient. Effective applications achieved  JR with significantly shorter JR-CL, but since the JR-CL correlates with the sinus CL, the JR-CL values in effective and non-effective applications overlap, depending on the sinus CL. The CL-ratio appears as an independent predictor of successful attempts, increasing the specificity of the JR and becoming an useful tool for identifying the effective applications. New studies involving larger numbers of patients are needed to confirm the value of our findings.

## Figures and Tables

**Table 1:**
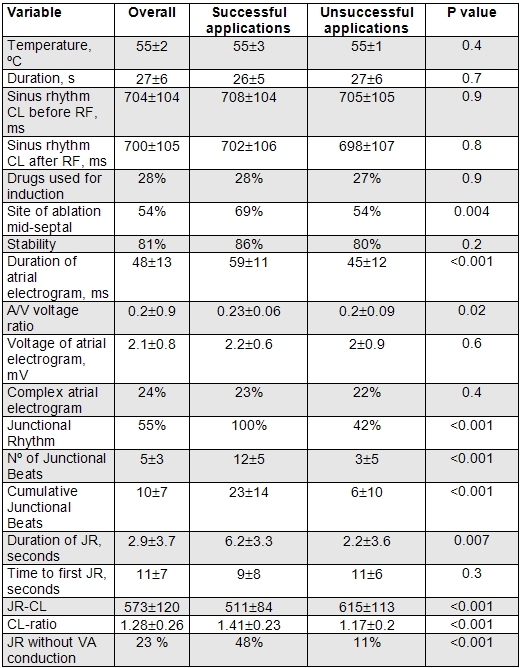
Characteristics of the applications of RF

RF: Radiofrequency Ablation. JR: Junctional Rhythm. JR-CL: Junctional Rhythm Cycle Length

**Table 2:**
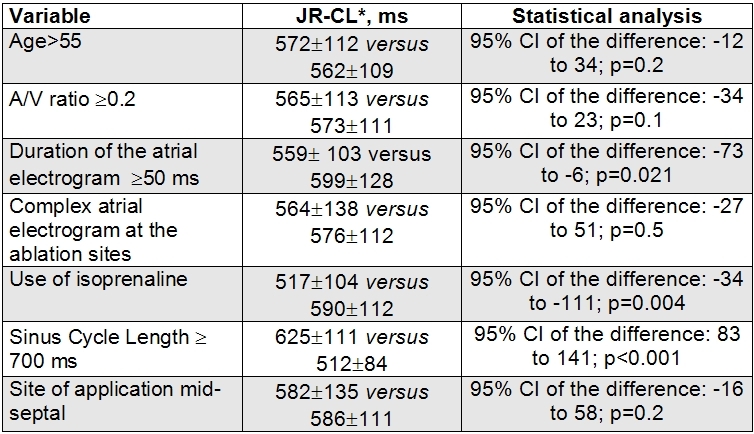
Comparison of the Junctional Rhythm Cycle Length in relation to several variables. Univariate analysis

RF: Radiofrequency Ablation. JR: Junctional Rhythm. JR-CL: Junctional Rhythm Cycle Length

**Table 3:**
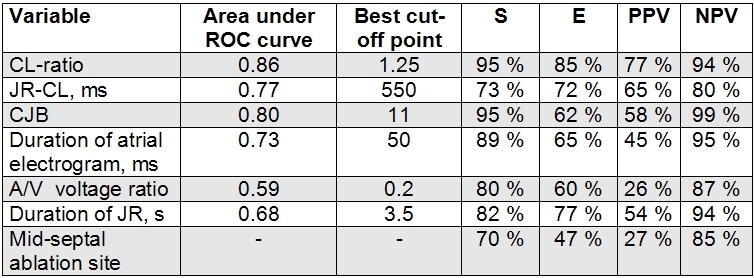
Statistical values for the probability of success of several variables

ROC curve:  Receiver-Operating Characteristic Curve. S: sensitivity. E: Specificity. PPV: Positive predictive value. NPV: Negative Predictive Value. JR-CL: Junctional Rhythm Cycle Length. CJB: Cumulative number of Junctional beats. JR: Junctional Rhythm

**Table 4:**
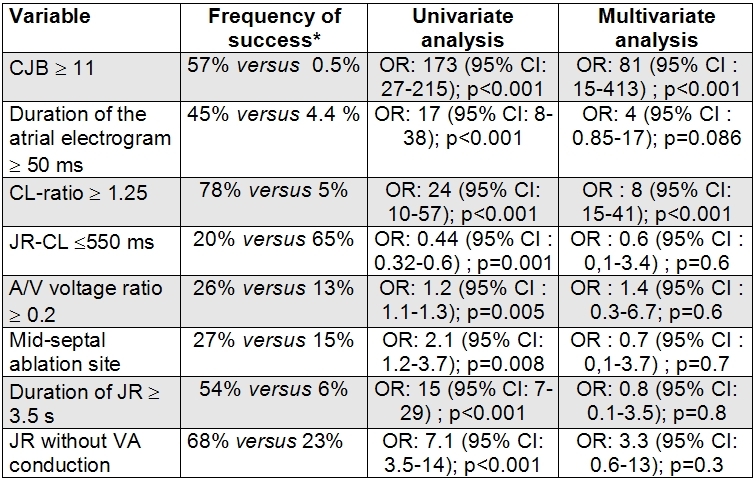
Statistical analysis of predictors of success

* Present versus absent. OD: Odds Ratio. CI: Confidence Interval. CJB: Cumulative number of Junctional beats. JR-CL: Junctional Rhythm Cycle Length. JR: Junctional Rhythm.

**Table 5:**
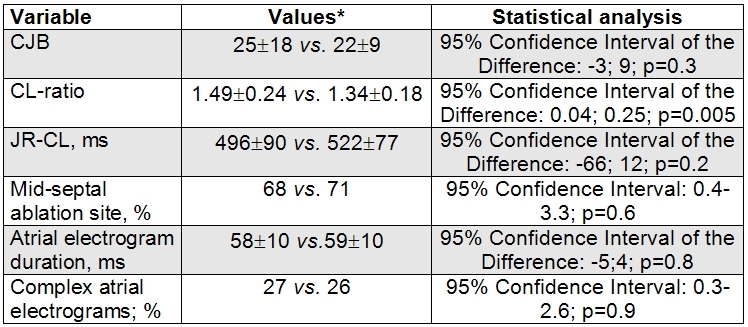
Relationship between the abolition of the dual AV nodal pathway physiology and different variables

* Present versus absent. OD: Odds Ratio. CI: Confidence Interval. CJB: Cumulative number of Junctional beats. JR-CL: Junctional Rhythm Cycle Length. JR: Junctional Rhythm
